# Applying the HIV Prevention Cascade to an Evaluation of a Large-Scale Combination HIV Prevention Programme for Adolescent Girls and Young Women in South Africa

**DOI:** 10.1007/s10461-023-04130-z

**Published:** 2023-07-18

**Authors:** Kate Bergh, Elona Toska, Zoe Duby, Darshini Govindasamy, Catherine Mathews, Tarylee Reddy, Kim Jonas

**Affiliations:** 1 Health Systems Research Unit, South African Medical Research Council, Francie Van Zijl Dr, Parrow Valley, Cape Town, 7501 South Africa; 2 Department of Psychology, University of Cape Town, Cape Town, South Africa; 3 Department of Sociology, Centre for Social Science Research, University of Cape Town, Cape Town, South Africa; 4 Department of Social Work and Social Development, University of Cape Town, Cape Town, South Africa; 5 Division of Social and Behavioural Sciences, School of Public Health and Family Medicine, University of Cape Town, Cape Town, South Africa; 6 Biostatistics Research Unit, South African Medical Research Council, Durban, South Africa

**Keywords:** HIV prevention cascades, Adolescent girls and young women, Condoms, PrEP, South Africa

## Abstract

Adolescent girls and young women (AGYW) in South Africa are at a three times higher risk of acquiring HIV than their male counterparts. The HIV prevention cascade is a tool which can be used to measure coverage of HIV prevention services, although there is limited empirical evidence to demonstrate its application in low-resourced settings. The unifying framework is a conceptualisation of the HIV prevention cascade which theorises that both motivation and access are required for an individual to effectively use an HIV prevention method. We applied this framework to data from a random sample of 127,951 beneficiaries of a combination HIV prevention programme for AGYW aged 15–24 in South Africa to measure the steps to, and identify key barriers to, effective use of male condoms and oral pre-exposure prophylaxis (PrEP) among this vulnerable population. Barriers to each step were analysed using univariate and multivariable logistic regression. Among self-reported HIV-negative AGYW who had sex in the past 6 months, effective use of condoms (15.2%), access to PrEP (39.1%) and use of PrEP (3.8%) were low. AGYW were: less likely to be motivated to use condoms if they believed that they had a faithful partner (aOR 0.44, 95% CI 0.22–0.90) or disliked condoms (aOR 0.26, 95% CI 0.11–0.57), less likely to access condoms if the place where AGYW accessed them was far away (aOR 0.25, 95% CI 0.10–0.64), more likely to effectively use condoms if they received counselling on how to use them (aOR 2.24, 95% CI 1.05–4.76), less likely to be motivated to use PrEP if they did not believe PrEP was efficacious (aOR 0.35, 95% CI 0.17–0.72), more likely to be motivated if they felt confident that they could use PrEP, and more likely to have access to PrEP if they had ever been offered PrEP (aOR 2.94, 95% CI 1.19–7.22). This combination HIV prevention programme and similar programmes should focus on risk-reduction counselling interventions for AGYW and their male partners to improve effective use of condoms and ensure easy access to condoms and PrEP by making them available in youth-friendly spaces. Our findings demonstrate that the application of HIV prevention cascades can inform AGYW HIV prevention programming in low-resourced settings.

**Supplementary Information:**

The online version contains supplementary material available at 10.1007/s10461-023-04130-z.

## Background

The United Nations Member States aim to achieve universal health coverage*,* reduce new HIV infections to fewer than 200,000 per year and meet the 95–95–95 treatment targets by 2030, in accordance with the Third Sustainable Development Goal (SDG 3) [1, 2]. However, there were an estimated 1.5 million new HIV infections globally in 2021 [3]. South Africa has the largest HIV epidemic in the world and accounted for 14% of these new infections [4]. New infections are approximately three times higher among adolescent girls and young women (AGYW) aged 15–24 in South Africa, compared to their male counterparts [5].

Factors which contribute to the high incidence of HIV among AGYW in South Africa include biomedical, behavioural and structural factors [6, 7]. Biomedical factors which make young women more susceptible to HIV infection include untreated sexually transmitted infections (STIs), an immature cervix, and abnormal bacterial flora [7]. Key behavioural factors include age-disparate sex, transactional sex (“the exchange of financial or material support, which occurs outside of the context of marriages, or ‘formal’ sex work” [8]) and gender-based violence (GBV) [7]. Structural barriers include economic and gender inequality as well as a lack of easily accessible youth-friendly sexual and reproductive health (SRH) services [6, 7, 9]. Women at a socio-economic disadvantage are more likely to engage in transactional sex, often in the context of age-disparate sexual relationships [10–12]. The power disparities in these types of relationships makes it difficult for young women to negotiate consistent condom use. In addition, AGYW are less likely to access HIV testing and prevention services than older age groups due largely to the inequitable social attitudes towards AGYW’s sexuality from family members, healthcare workers and their communities [13–15].

The *UNAIDS Decision Making Aide for Investments into HIV Prevention Programmes for AGYW 2023* recommends a combination of behavioural, biomedical, and structural interventions to prevent new infections among young women [16]. In areas with high HIV infection, basic packages including access to education, economic empowerment, youth-friendly SRH education and services, HIV testing and treatment, male and female condoms, pre-exposure prophylaxis (PrEP) and risk-reduction counselling are recommended, as well as additional services such as HIV testing for male partners. In South Africa, the main biomedical interventions available for HIV-negative AGYW who have sex include condoms and oral PrEP. Daily oral PrEP is recommended for AGYW at risk of HIV acquisition as it is a medication that can prevent HIV acquisition and empowers young women to make decisions about HIV prevention that do not require partner approval [17]. For HIV prevention programmes to be successful, they need to provide quality care to populations most at risk of HIV infection with high intensity and at scale [18].

Health service-coverage cascades have been proposed as the most appropriate way to measure effective coverage of health products and services and progress towards universal health coverage [19]. Effective coverage is defined as the proportion of the population in need of a service that experienced the service at a level of care sufficient to achieve positive health outcomes of which individual uptake and adherence to health products and services is a critical component. The HIV prevention cascade is a promising new framework for measuring coverage of HIV prevention interventions and services, and related barriers, which can help programmes to set targets and adapt and scale interventions [18, 20]. HIV prevention cascades follow a similar logic to the HIV treatment cascade which describes the steps that a person living with HIV needs to take from HIV diagnosis to achieve viral suppression [21]. However, the HIV prevention cascade measures the steps required by individuals in a population at risk of HIV infection to achieve effective use of a prevention method and prevent infection. The HIV prevention cascade is more complicated because the population in need of HIV prevention can change over time and there are different types of HIV prevention methods [18]. A simple HIV prevention cascade which can be applied in different contexts could promote comparability across populations, geographic areas and prevention methods [20].

Several prevention cascades were developed in a brainstorming session at a UNAIDS workshop (December 2016); however, most were designed for high-resourced health systems with access to sophisticated data systems [22–24]. Only a few prevention cascades have been proposed which are also applicable in low-resourced settings, including the unifying framework, the user- and provider-centric cascades, and the basic and expanded models [18, 20, 25]. We used the unifying framework, which follows the same steps as the expanded model, as it proposes one simple model for users of the prevention method and does not include “infections prevented” as a step which requires mathematical modelling to measure [20].

**Fig. 1:**
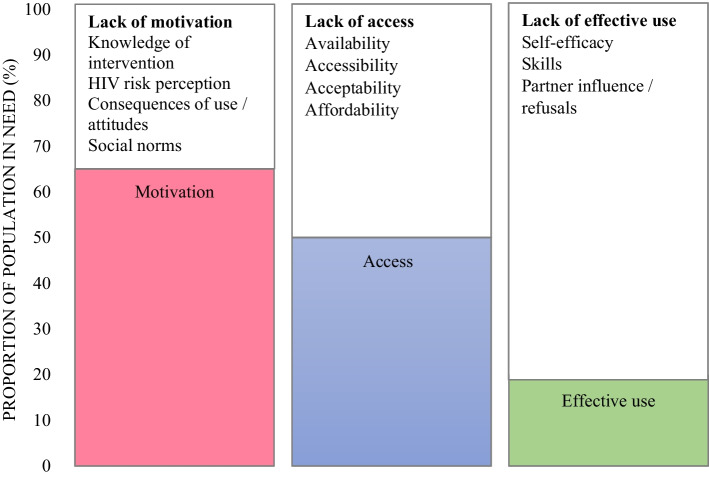
An adapted unifying framework describing motivation, access and effective use of HIV prevention methods and the broad barriers to each step of the cascade, based on Schaefer et al. (2019)’s model, and including attitude as a barrier to motivation, based on Fisher et al. (2006)’s definition of personal motivation [20, 27].

The unifying framework is a three-step cascade which includes motivation to use, access to and effective use of HIV prevention methods among a population in need of HIV prevention (Fig. 1) [20]. Motivation is the cognitive process that leads to behavioural intent and the desire to use a particular prevention method. Motivation can only translate into the action of using condoms or PrEP if there is access to the HIV prevention method. Individuals with both motivation and access can effectively use the prevention method, which is defined as the uptake and adherence to an intervention that is required to achieve the maximum protection from HIV infection. The unifying framework also proposes broad barriers to motivation, access and effective use which are described in Fig. 1 [20, 22, 26]. We have added “attitudes” to the broad barrier called “consequences of use” to fully capture the definition of personal motivation as it is described in the Information-Motivation-Behaviour skills model [27].

Limited empirical research has applied the various HIV prevention cascades to data from low-resourced countries [20, 25, 26, 28–31]. Our study was guided by three papers which applied the unifying framework and a similar cascade to data on condom and PrEP use in Zimbabwe using separate and combined cascades [20, 26, 28]. We aimed to measure the steps of the HIV prevention cascade for male condoms and oral PrEP, and identify key barriers to each step, among a random sample of 127,951 AGYW beneficiaries from 6 districts in South Africa with high HIV incidence who were enrolled in one of the largest combination HIV prevention programmes in the country, to demonstrate how HIV prevention cascades can be applied to programmatic data to inform interventions.

## Methods

In this study, we applied the unifying framework to data from a mixed-methods process evaluation (HERStory2), conducted by the South African Medical Research Council (SAMRC), of the first two years of the My Journey programme (Grant Period: 2019–2022), funded by the Global Fund to Fight AIDS, Tuberculosis and Malaria [32]. The objective of the process evaluation was to determine whether the coverage of the programme was aligned to the programme targets and theory of change. The process evaluation was conducted during the second wave of COVID-19 and lockdowns in South Africa. As a result, data collection took place telephonically.

### The My Journey Programme

The My Journey programme 2019–2022 was a large-scale combination HIV prevention programme for AGYW aged 15–24 in 12 districts in South Africa in which AGYW are at the highest risk of HIV infection [33]. The baseline evaluation of this programme, which was conducted among 4,399 AGYW aged 15–24 in 6 of the 12 programme districts (three were the same as those sampled for the process evaluation), reported an HIV prevalence of 12.4% [8]. Among participants who had ever had sex (n = 3009), 15.9% of participants were living with HIV.

The programme aimed to reduce HIV infection, teenage pregnancy and GBV, and increase retention in school and access to economic opportunities. Implementation was managed by three non-governmental organisations. The programme offered two main service components: (1) *Core Services*, usually offered first, included a risk assessment and provision of optional HIV testing, condoms, and health information relating to HIV, TB, STIs and GBV; (2) *Layered Services* were additional biomedical, behavioural and structural services offered over time depending on the needs of the beneficiary. Biomedical interventions included the promotion of long-acting reversible contraceptives, easy access to quality condoms and information on how to use them, and PrEP demand creation and provision for AGYW at risk of HIV. Behavioural interventions included a teen parenting programme, psychosocial support, peer education and comprehensive sexuality education. Structural interventions included access to safe spaces, self-defence classes, dialogue with men to change social norms, academic support, career guidance, access to work opportunities, and provision of dignity packs. Services were provided at schools, colleges, mobile clinics and safe spaces in the community, and externally, through referrals to government services.

The programme was built upon the theory of change model which reads as follows: “IF adolescent girls and young women are identified through various entry points (in schools, communities through NGOs, churches, public spaces and higher education institutions through TVET colleges) and have their risks and vulnerabilities assessed and, IF AGYW are linked to biomedical, behavioural and structural HIV prevention interventions, THEN that may lead to positive heath and behavioural outcomes, that, in turn should lead to reductions in new HIV infection among this group, IF programmatic, financial and political assumptions hold true” (extracted from AGYW Programme Description) [32]. The theory of change is closely aligned with the concept of effective coverage which can be measured using health service-coverage cascades such as the HIV prevention cascade.

### Sample and Data

A descriptive cross-sectional survey was carried out between December 2020 and February 2021 with AGYW beneficiaries in 6 of the 12 districts across 6 provinces. The 6 districts were selected in consultation with implementers to represent both urban and rural districts, and the different implementers. The sampling frame for this study was a de-identified record of all programme beneficiaries (127,951 AGYW). Beneficiaries were stratified by district and age group (15–19 vs. 20–24), and for the younger age group, by whether they reported being in school. Twice as many beneficiaries in the younger age group were sampled under the assumption that 50% of these beneficiaries would not yet have had sex and not contribute to measures relating to PrEP and contraceptive usage. We randomly sampled 2,160 beneficiaries (360 per district) from the stratified record of all beneficiaries who had been enrolled in the programme for at least one year to ensure that they had had time to participate in the programme.

The SAMRC provided the combination prevention programme implementers with a list of unique identifiers for sampled AGYW. Implementers contacted AGYW on the list and, using a script, provided details about the study and asked if AGYW would be willing to be contacted by the research team to be invited to participate in the study.

### Ethical Considerations

Ethics approval for this study was granted by the SAMRC Research Ethics Committee (EC036-9/2020). An informed consent process was conducted telephonically with all participants prior to the survey. If participants were under 18 years old, consent was first obtained from a parent or legal guardian and then the AGYW beneficiary. Verbal consent was recorded, and the audio-recording was saved on a password protected computer. Participants could conduct the survey in their language of choice. Each participant received ZAR 100.00 (± US$ 6.00) reimbursement for their time.

### Measures

Binary and categorical variables relating to socio-demographic characteristics, sexual behaviour and use of HIV prevention methods were created from the survey questions (supplementary information). A categorical variable was created for relative socio-economic status (SES) using 13 SES-related questions in the survey (supplementary information). The SES variable was created through a cluster analysis with the K-Modes algorithm [34], using the “klaR” package [35]. The variable NEET (not in education, employment or training) was created from 6 questions on educational and employment status during 2020 (supplementary information).

For the HIV prevention cascade, we defined and measured the population in need, motivation, access and effective use of male condoms and PrEP. These definitions were informed by a stakeholder workshop on HIV prevention cascades and published work by Moorhouse et al. (2019) (Table 1) [22, 26]. However, the definition for the population in need was also influenced by findings from the baseline evaluation of the My Journey programme which found that HIV prevalence among AGYW aged 15–24 was very high and even higher among AGYW who had ever had sex [8, 33]. Barriers to these steps were selected and categorised based on the broad barriers described in Fig. 1 and are described in Tables 3 and 4.

**Table 1:** Definitions for the indicators of the population in need, motivation, access and effective use of male condoms and PrEP

Domain	Male condoms	PrEP
Population in need	AGYW who reported that they were HIV-negative and had “penile-vaginal” or “penile-anal” sex in the past 6 months	AGYW who reported that they were HIV-negative, had “penile-vaginal” or ‘penile-anal” sex within the past 6 months , and had never taken PrEP
Motivation	AGYW reported that she *definitely* or *probably* wanted to use male condoms if they were freely available	AGYW reported that she *definitely* or *probably* wanted to use PrEP if it was freely available
Access	AGYW reported that she found it *easy* or *very easy* to get male condoms	AGYW reported that she found it *easy* or *very easy* to get to a place where PrEP is provided
Effective use	AGYW reported that she used condoms *100%* of the time when she had sex with her last male partner	Not applicable

Both cascades cover a 6-month period during which the population in need reported having “penile-vaginal” or “penile-anal” sex and is thus at risk of HIV acquisition. This timeframe allows for comparability with other cascades and minimizes recall bias [18]. For the condom cascade, we focus on male condoms, although the indicator for effective use does not specify whether the participant used male or female condoms, but since only 2% of the population in need had used a female condom in the past 6 months, the results are still specific to male condoms. For the PrEP cascade, we did not look at effective use of PrEP as PrEP was still being rolled out during the survey; only 3.8% of HIV-negative AGYW who had sex in the past 6 months were on PrEP and only participants who had never taken PrEP were asked about motivation and access.

Post-analysis sample weights were applied to the survey data based on sample realisation in three groups (AGYW 15–19 in-school, 15–19 out-of-school and 20–24). Sample weights were applied to descriptive statistics and cascade indicators to provide insights into characteristics of AGYW involved in the broader AGYW programme, but as design-based weighting was not used, it was not necessary to apply sample weights to our analyses, although they are provided in the supplementary information [36].

### Statistical Analysis

Statistical analyses were conducted in Stata (Stata SE 17.0, StataCorp, Texas, USA). HIV prevention cascades were created for male condoms and PrEP based on Moorhouse et al. (2019) and Schaeffer et al. (2019)’s methodology [20, 26]. The population in need is the denominator for each bar of the cascade. The numerator for each bar of the cascade includes only participants in the population in need who were included in the previous bar; the same approach was also adopted for the analysis of barriers to each step of the cascade.

Using logistic regression, univariate and multivariable analyses were conducted to identify the barriers to each step of the cascade for condoms and PrEP [26, 28]. For the multivariable analyses, a forward stepwise regression analysis was conducted with a threshold of 0.10 to see which barriers were independently associated with each step of the cascade [37]. Collinear variables were removed through the forward stepwise regression.

## Results

### Sample Realisation

Of the 2,160 participants randomly sampled for this study, 515 AGYW participated in the survey. Sample realisation was 23.8% (supplementary information).

### Description of Sample

Table 2 describes AGYW characteristics among a sub-sample of AGYW who reported that they were HIV-negative and had sex in the past 6 months (n = 301). The majority (58.5%) of these participants were in the 20–24 age group. The proportion of AGYW who were NEET in 2020 was 20.0% in the older and 8.3% in the younger age group. Over a third (38.8%) of AGYW had ever been pregnant. Very few (5.6%) participants reported transactional sex (that they had oral/anal/vaginal sex to pay for things they needed), but 24.6% of AGYW had a male sex partner who was five or more years older, in the past 6 months.

**Table 2:** AGYW characteristics relating to HIV risk among AGYW who were HIV-negative and had sex in the past 6 months, stratified by age group (weighted) (n = 301)

Variable	Age group N (%)		
	Total	15–19	20–24
Age group (n = 301)	–	125.0 (41.5)	176.0 (58.5)
Lowest SES group out of four categories (n = 300)	60.1 (20.0)	22.0 (20.6)	34.6 (17.9)
NEET in 2020 (n = 301)	42.3 (14.0)	8.9 (8.3)	38.7 (20.0)
Orphan (both parents are deceased or missing) (n = 301)	38.9 (12.9)	13.5 (12.5)	23.6 (12.2)
AGYW has ever had an HIV test (n = 301)	292.6 (97.2)	102.5 (94.9)	192.3 (99.6)
AGYW has ever been pregnant (n = 301)	116.8 (38.8)	21.6 (20.0)	99.4 (51.5)
AGYW had more than one boyfriend or male sex partner in the past 6 months (n = 300)	68.6 (22.9)	21.2 (19.7)	51.1 (26.6)
AGYW reported having a male sex partner who was five or more years older than her in the past 6 months (n = 301)	74.0 (24.6)	22.1 (20.5)	56.3 (29.2)
AGYW had oral, anal, or vaginal sex to pay for things she needed in the past 6 months (n = 301)	16.8 (5.6)	6.1 (5.7)	12.9 (6.7)

**Fig. 2:**
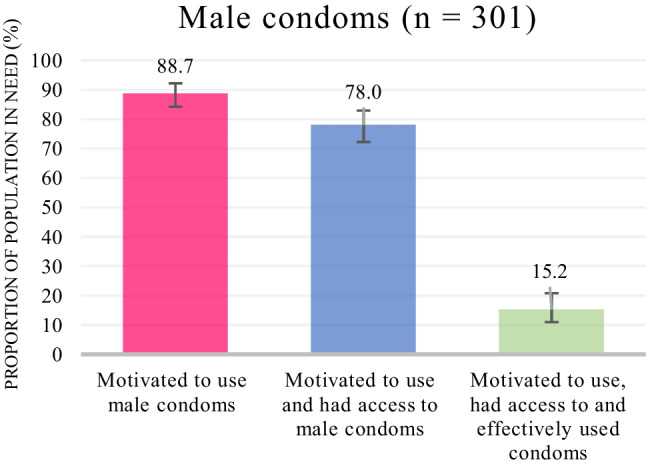
Weighted HIV prevention cascade describing motivation, access and effective use of male condoms among AGYW who were HIV-negative and had sex in the past 6 months

**Fig. 3:**
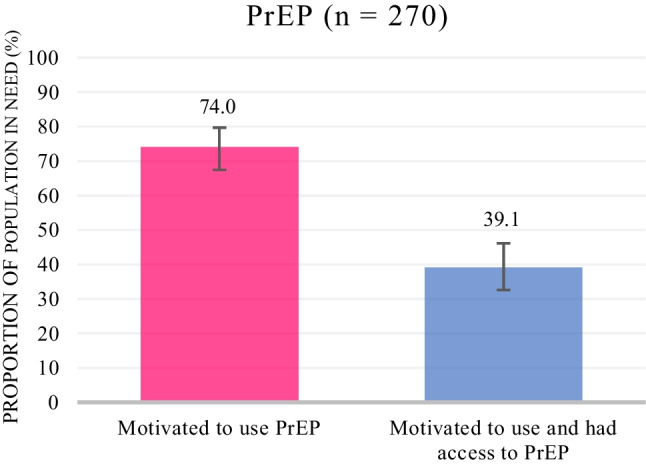
Weighted HIV prevention cascade describing motivation and access to PrEP among AGYW who were HIV-negative, had sex in the past 6 months and had never taken PrEP

Figures 2 and 3 depict the HIV prevention cascades for male condoms and PrEP, respectively. Among AGYW who were HIV-negative and had sex in the past 6 months: 88.7% (95% CI 84.2–92.1%) were motivated to use male condoms; 78.0% (95% CI 72.3–82.9%) were motivated and had access; and 15.2% (95% CI 10.9–20.8%) were motivated, had access and effectively used condoms. Among AGYW who were HIV-negative, had sex in the past 6 months and had never taken PrEP, 74.0% (95% CI 67.5–79.7%) were motivated to use PrEP and 39.1% (95% CI 32.6–46.1%) were motivated and had access to PrEP.

Table 3 describes the factors associated with motivation, access and effective use of male condoms. In multivariable analyses, participants were less likely to be motivated to use condoms if they believed that they had one faithful partner (aOR 0.44, 95% CI 0.22–0.90) or did not like using condoms (aOR 0.26, 95% CI, 0.11–0.57), participants were less likely to have access to condoms if the place where they accessed condoms was far away (aOR 0.25, 95% CI 0.10–0.64), and participants were more likely to effectively use condoms if they had had instructions or counselling on how to use male condoms (aOR 2.24, 95% CI 1.05–4.76). In univariate analyses, participants were less likely to effectively use condoms if they had a partner who was five or more years older in the past 6 months (OR 0.40, 95% CI 0.16–1.01).

**Table 3:** Univariate and multivariable analyses of factors associated with motivation to use, access to and effective use of male condoms

Among AGYW who were HIV-negative and had sex in the past 6 months (n = 301)			
Barrier	Motivated to use male condoms N (%)	Crude odds ratio (OR) (95% CI)	Adjusted odds ratio (aOR) (95% CI)
Age group (n = 301)			
15–19	92 (85.2)	–	–
20–24	169 (87.6)	1.22 (0.62–2.42)	
Relative SES group (out of four levels) (n = 300)			
	–	0.95 (0.69–1.30)	–
AGYW NEET in 2020 (n = 301)			
No	227 (87.3)	–	–
Yes	34 (82.9)	0.71 (0.29–1.72)	
Knowledge of intervention			
AGYW did not think that male condoms reduce an HIV-negative person’s risk of getting HIV by 70% or more when they have sex with someone who has HIV (n = 298)			
Believed	176 (87.6)	–	–
Did not believe	82 (84.5)	0.78 (0.39–1.55)	
HIV risk perception			
AGYW did not think she was at risk of getting HIV (n = 301)			
No	255 (87.0)	–	–
Yes	6 (75.0)	0.45 (0.09–2.30)	
AGYW believed she had one faithful partner (n = 301)			
No	194 (90.2)	–	–
Yes	67 (77.9)	0.38 (0.19–0.75)**	0.44 (0.22–0.90)*
Consequences of use/attitudes			
AGYW does not like using condoms (n = 301)			
No	236 (89.7)	–	–
Yes	25 (65.8)	0.22 (0.10–0.48)**	0.26 (0.11–0.57)**
AGYW agrees or strongly agrees that if she asked her current or most recent male partner to use a condom, he would get angry (n = 300)			
Disagree	224 (86.8)	–	–
Agree	36 (85.7)	0.91 (0.36–2.32)	
AGYW is embarrassed to get male condoms (n = 301)			
No	152 (88.9)	–	–
Yes	109 (83.8)	0.65 (0.33–1.26)	
AGYW is worried someone will see her getting male condoms (n = 301)			
No	163 (87.6)	–	–
Yes	98 (85.2)	0.81 (0.41–1.60)	

Among AGYW who were motivated to use male condoms, HIV-negative and had sex in the past 6 months (n = 260)			
Barrier	Had access to male condoms N (%)	Crude odds ratio (OR) (95% CI)	Adjusted odds ratio (aOR) (95% CI)
Age group (n = 260)			
15–19	76 (82.6)	–	–
20–24	152 (90.5)	2.00 (0.95–4.22)	2.33 (0.95–5.70)
Relative SES group (out of four levels) (n = 260)			
	–	1.14 (0.81–1.62)	–
AGYW NEET in 2020 (n = 260)			
No	199 (88.1)	–	–
Yes	29 (85.3)	0.79 (0.28–2.21)	
Availability			
AGYW did not have condoms (n = 260)			
No	175 (86.6)	–	–
Yes	53 (91.4)	1.64 (0.60–4.46)	
AGYW reported that there was a stock-out and they did not have condoms for her (n = 260)			
No	224 (88.2)	–	–
Yes	4 (66.7)	0.27 (0.05–1.53)	
Accessibility			
AGYW was sometimes or often unable to get male condoms because of COVID-19 or the lockdown (n = 211)			
No	133 (89.9)	–	–
Yes	55 (87.3)	0.78 (0.31–1.93)	
In the past month, someone from an organisation involved in this research has provided the AGYW with condoms or linked her to people who could provide them (n = 260)			
No	188 (87.0)	–	–
Yes	40 (90.9)	1.49 (0.49–4.48)	
The place where AGYW gets her condoms was not open when she had free time (n = 260)			
No	217 (88.9)	–	–
Yes	11 (68.8)	0.27 (0.09–0.85)*	
The place where AGYW gets her condoms is far away (n = 260)			
No	194 (90.2)	–	–
Yes	34 (75.6)	0.33 (0.15–0.76)**	0.25 (0.10–0.64)**
Acceptability			
AGYW finds it difficult to get male condoms because of the lack of privacy and confidentiality when getting them (n = 260)			
No	181 (89.6)	–	–
Yes	47 (81.0)	0.50 (0.22–1.10)	
AGYW reported that the negative attitudes of health workers who give her condoms is a barrier (n = 260)			
No	219 (88.3)	–	–
Yes	9 (75.0)	0.40 (0.10–1.55)	
Affordability			
AGYW finds it difficult to get male condoms because it is expensive to get them (n = 260)			
No	217 (88.6)	–	–
Yes	11 (73.3)	0.35 (0.11–1.19)	

Among AGYW who had access to male condoms, were motivated to use male condoms, were HIV-negative and had sex in the past 6 months (n = 223)			
Barrier	Effectively used condoms N (%)	Crude odds ratio (OR) (95% CI)	Adjusted odds ratio (aOR) (95% CI)
Age group (n = 223)			
15–19	15 (20.0)	–	–
20–24	27 (18.2)	0.89 (0.44–1.80)	
Relative SES group (out of four levels) (n = 223)			
	–	1.10 (0.81–1.51)	–
AGYW NEET in 2020 (n = 223)			
No	36 (18.5)	–	–
Yes	6 (21.4)	1.20 (0.46–3.19)	
AGYW has 6 or more drinks on one occasion every month or more frequently (n = 223)			
No	24 (17.6)	–	–
Yes	17 (19.8)	1.15 (0.58–2.29)	
Skills			
AGYW has had instructions or counselling on how to use male condoms (n = 223)			
No	13 (14.1)	–	–
Yes	29 (22.1)	1.73 (0.84–3.54)	2.24 (1.05–4.76)*
Partner influence/refusals			
AGYW’s sexual partner does not want her to use condoms (n = 223)			
No	41 (20.7)	–	–
Yes	1 (4.0)	0.16 (0.02–1.21)	0.18 (0.02–1.38)
In the past 6 months , AGYW had sex (oral, vaginal or anal) with someone to pay for the things she needs (n = 223)			
No	42 (19.7)		
Yes	0.0 (0.0)	Omitted	Omitted
In the past 6 months, AGYW had sex with a man who was older than her by 5 years or more (n = 223)			
No	36 (22.0)	–	–
Yes	6 (10.2)	**0.40 (0.16–1.01)**	0.41 (0.16–1.06)
In the past 6 months, AGYW was afraid of her partner (n = 223)			
Less often or never	40 (19.3)	–	–
More than once	2 (12.5)	0.60 (0.13–2.73)	

Bold = p value $\le$ 0.05; *p value < 0.05; **p value < 0.01; Omitted indicates exposed group was too small for variable to be included in logistic regression

Table 4 describes the factors associated with motivation and access to PrEP. In multivariable analysis, participants were less likely to be motivated to use PrEP if they did not believe that PrEP could reduce a person’s risk of getting HIV by 70% or more (aOR 0.35, 95% CI 0.17–0.72) and more motivated to use PrEP if they were NEET in 2020 (aOR 4.60, 95% CI 1.15–18.35), reported being worried about people thinking they had HIV when getting PrEP (aOR 2.28. 95% CI 1.03–5.05) with weak significance and felt confident that they could use PrEP in the correct way and despite what others think. For access, participants were more likely to have access to PrEP if they had ever been offered PrEP in multivariable analysis (aOR 2.94, 95% CI 1.19–7.22).

**Table 4:** Univariate and multivariable analyses of factors associated with motivation to use and access to PrEP

Among AGYW who were HIV-negative, had sex in the past 6 months and have never taken PrEP (n = 270)			
Barrier	Motivated to use PrEP N (%)	Crude odds ratio (OR) (95% CI)	Adjusted odds ratio (aOR) (95% CI)
Age group (n = 270)			
15–19	74 (74.7)	–	–
20–24	131 (76.6)	1.11 (0.62–1.97)	
Relative SES group (out of four levels) (n = 270)			
	–	1.14 (0.87–1.49)	–
AGYW NEET in 2020 (n = 270)			
No	176 (75.2)	–	–
Yes	29 (80.6)	1.37 (0.57–3.28)	4.60 (1.15–18.35)*
Knowledge of intervention			
AGYW knew about PrEP and was sure about what it was (n = 270)			
No	131 (77.1)	–	–
Yes	74 (74.0)	0.85 (0.48–1.50)	0.51 (0.24–1.06)
AGYW believed that PrEP could reduce a person’s risk of getting HIV by 70% or more (n = 260)			
Believed	139 (84.8)	–	–
Did not believe	60 (62.5)	0.30 (0.17–0.54)**	0.35 (0.17–0.72)**
HIV risk perception			
AGYW did not think she was at risk of getting HIV (n = 270)			
No	201 (76.1)	–	–
Yes	4 (66.7)	0.63 (0.11–3.50)	
AGYW has one faithful partner who she trusts (n = 270)			
No	141 (73.8)	–	–
Yes	64 (81.0)	1.51 (0.79–2.89)	
In the past 6 months , AGYW had sex (oral, vaginal or anal) with someone to pay for the things she needed (n = 270)			
No	195 (75.3)	–	–
Yes	10 (90.9)	3.28 (0.41–26.14)	
In the past 6 months, AGYW had sex with a man who was older than her by 5 years or more (n = 270)			
No	151 (75.9)	–	–
Yes	54 (76.1)	1.01 (0.54–1.90)	
In the past 6 months, AGYW was afraid of her partner (n = 270)			
Less often or never	189 (75.9)	–	–
More than once	16 (76.2)	1.02 (0.36–2.89)	
AGYW had an HIV test in the past 6 months (n = 257)			
No	40 (76.9)	–	–
Yes	157 (76.6)	0.98 (0.48–2.02)	
Consequences of use/attitudes			
AGYW reported that it is difficult to get to a place to get PrEP because she worries about people thinking she is HIV-positive (n = 270)			
No	130 (72.6)	–	–
Yes	75 (82.4)	1.77 (0.94–3.32)	2.28 (1.03–5.05)*
AGYW was confident she would be able to use PrEP if she wanted to (n = 270)			
No	37 (53.6)	–	–
Yes	168 (83.6)	4.40 (2.41–8.04)**	2.99 (1.41–6.35)**
AGYW was confident she would be able to take PrEP every day (n = 270)			
No	48 (58.5)	–	–
Yes	157 (83.5)	3.59 (2.00–6.43)**	
AGYW was confident she would always be able to take PrEP after a meal (n = 270)			
No	43 (56.6)	–	–
Yes	162 (83.5)	3.89 (2.15–7.02)**	3.04 (1.37–6.75)**
AGYW was confident she would be able to use PrEP if she had to hide it from her partner (n = 270)			
No	104 (72.2)	–	–
Yes	101 (80.2)	1.55 (0.88–2.75)	
AGYW was confident she would be able to use PrEP if her friends disapproved of it (n = 270)			
No	34 (59.6)	–	–
Yes	171 (80.3)	2.75 (1.47–5.16)**	
AGYW was confident she would be able to use PrEP if her parents and family elders disapproved (n = 270)			
No	52 (61.2)	–	–
Yes	153 (82.7)	3.03 (1.70–5.41)**	1.93 (0.92–4.02)
AGYW was confident she would be able to use PrEP if people thought she had HIV (n = 270)			
No	26 (54.2)	–	–
Yes	179 (80.6)	3.52 (1.82–6.80)**	

Among AGYW who were motivated to use PrEP, were HIV-negative, had sex in the past 6 months and had never taken PrEP (n = 205)			
Barrier	Had access to PrEP N (%)	Crude odds ratio (OR) (95% CI)	Adjusted odds ratio (aOR) (95% CI)
Age group (n = 205)			
15–19	35 (47.3)	–	–
20–24	78 (59.5)	1.64 (0.92–2.91)	
Relative SES group (out of four levels) (n = 205)			
	–	0.91 (0.70–1.18)	–
AGYW NEET in 2020 (n = 205)			
No	93 (52.8)	–	–
Yes	20 (69.0)	1.98 (0.86–4.60)	
Availability			
AGYW has ever been offered PrEP (n = 205)			
No	91 (51.7)	–	–
Yes	22 (75.9)	2.94 (1.19–7.22)*	2.94 (1.19–7.22)*
Accessibility			
AGYW believes the opening hours of the PrEP clinic/service would not suit her (n = 205)			
No	105 (55.6)	–	–
Yes	8 (50.0)	0.80 (0.29–2.22)	
AGYW believes it is far to go to the PrEP clinic/service (n = 205)			
No	102 (55.1)	–	–
Yes	11 (55.0)	0.99 (0.39–2.51)	
Acceptability			
AGYW would worry about lack of privacy or confidentiality at a PrEP clinic/service (n = 205)			
No	84 (55.6)	–	–
Yes	29 (53.7)	0.93 (0.50–1.73)	
AGYW believes that the negative attitudes of the health workers at a PrEP clinic/service would make it difficult for her to get PrEP (n = 205)			
No	86 (52.8)	–	–
Yes	27 (64.3)	1.61 (0.80–3.25)	
Affordability			
AGYW believes it would cost too much to get to the clinic/service to get PrEP (n = 205)			
No	107 (54.9)	–	–
Yes	6 (60.0)	1.23 (0.34–4.51)	

Bold = p value ≤ 0.05; *p value < 0.05; **p value < 0.01

## Discussion

We aimed to measure the steps of the HIV prevention cascade and identify barriers to these steps to demonstrate how the HIV prevention cascade can be applied to programmatic data from low-resourced settings to inform interventions and improve condom and PrEP uptake and adherence within the My Journey programme and other combination HIV prevention programmes in South Africa. Results were interpreted in the context of the second wave of COVID-19 in South Africa.

Despite COVID-19 restrictions, motivation (89%) and access (78%) to male condoms was high. This is not surprising as condoms are an established HIV prevention method and are widely available in South Africa. Results on access to condoms were consistent with the nationally representative NIDS-CRAM study which conducted telephonic interviews with individuals aged 15–49 during the first wave of COVID-19 in South Africa and found that 22% could not access condoms [38]. Effective use of condoms was considerably lower (15%), but comparable to consistent condom use among women aged 15–49 in South Africa reported as 16% in the nationally representative demographic and health survey in 2016 [39]. One would expect access and effective use within the My Journey programme to be higher than the national average if targets were being achieved, but these similar findings could be because the evaluation took place during the early stages of the programme’s implementation.

Our study identified the independent barriers to condom use as disliking condoms and believing that you had a faithful partner (motivation), and distance to facilities (access). Receiving counselling on how to use condoms was a facilitator of effective use. In addition, 25% of the population in need reported having an age-disparate relationship, although this had only a weakly negative association with effective use in univariate analysis. These findings are aligned with a narrative systematic review of 23 qualitative studies on the determinants of condom use among adolescents in Southern Africa [40]. Some of the key themes outlined in the review were “restrictive masculinities favouring male sexual decision-making and stigmatising condom use in committed relationships”, unequal power dynamics in sexual relationships and negative attitudes towards condom use among adolescents. The baseline evaluation of the My Journey programme had similar findings which described how AGYW believed that condomless sex demonstrated love and commitment, had negative beliefs about condoms and their side effects, and had a fear of violent reactions from partners when the topic of condom use was raised [41]. Young men were motivated to have condomless sex by a desire for respect and masculine sexual maturity.

A systematic review of 292 reviews/primary studies on the effectiveness of HIV prevention interventions suggests that the most effective interventions for demand-side (motivation) barriers to condom use are peer-led information, education and communication interventions combined with direct provision of condoms [42]. Interventions to improve effective use included individual, couple or group risk-reduction counselling to improve self-efficacy and skills, and livelihood strengthening to empower AGYW to choose their sexual partners. The UNAIDS also recommends these interventions for AGYW at high risk of acquiring HIV as well as the inclusion of male partners in risk-reduction counselling [16]. Since motivation was relatively high and peer education was already provided through the My Journey programme, we suggest that the programme focuses on risk-reduction counselling for AGYW and their partners to improve effective use of condoms, supported by our finding that receiving counselling about condom use increased effective condom use [33]. Findings from the baseline evaluation of the My Journey programme also recommend interventions which teach communication and negotiation skills so that AGYW can transfer their knowledge of safe sex practices to their partners [41]. To address the prevalence of age-disparate sexual relationships, the My Journey programme should consider scaling up economic strengthening interventions such as academic support, career guidance and access to work opportunities to prevent AGYW from engaging in transactional sex within these relationships which could affect their ability to negotiate condom use [8, 33].

In terms of access, the barriers to accessing condoms during this study may have been influenced by the COVID-19 pandemic as some non-essential health facilities reduced their operating hours and public transport was difficult due to social distancing. Thus, we recommend the mass distribution of condoms through schools, places of work, mobile clinics, safe spaces and community centres in pandemic situations [38]. Programmes should engage with schools on the benefits of condom provision at school to ensure permission for this type of intervention is granted.

In terms of PrEP, motivation to use PrEP was high (74%). Independent barriers to motivation included not believing that PrEP could reduce HIV risk while facilitators included being NEET and having the confidence to use PrEP in the correct way and despite what others may think. These findings were similar to those of a qualitative study among males and females aged 13–24 in Uganda, Zimbabwe and South Africa on the barriers/facilitators to PrEP uptake [43]. This study found that participants expressed a willingness to take PrEP but were constrained by scepticism about the medication’s effectiveness, the complex timing of administration, fear of HIV-related stigma, and parents finding out they were sexually active. We also had a contradictory finding that participants were more likely to be motivated to use PrEP if they worried that people may think they were living with HIV if they went to get PrEP. However, this could be because AGYW who were more motivated to get PrEP had considered these challenges more carefully. Nevertheless, findings from the qualitative component of the HERStory 2 process evaluation highlight PrEP stigma related to associations with antiretrovirals and promiscuity as major barriers to PrEP acceptability [44]. The positive relationship between motivation and NEET is also interesting and may be because AGYW who are NEET are more likely to engage in transactional sex and age-disparate relationships where condom use is difficult to negotiate, making PrEP an appealing HIV prevention method [10, 12].

Peer and community-led education campaigns for AGYW, parents, partners and community members are recommended to increase knowledge and uptake of PrEP among AGYW and sensitise communities to PrEP [43, 45, 46]. Demand creation interventions for PrEP and awareness raising in schools, communities and health facilities were meant to be provided through the programme, but as PrEP had not been fully implemented by the programme at the time of the study and implementation had been challenging partly due to COVID-19 restrictions, not all participants may have benefited from these interventions, and this should be a focus of the programme in the next grant period [32, 33].

Access to PrEP (39%) was much lower than access to condoms (78%) even if we add the 3.8% of the population in need who were already on PrEP and not included in the cascade. Never being offered PrEP was the only barrier to access. This finding is again explained by the challenges in PrEP implementation during COVID-19 lockdowns [32]. In consultation with programme implementers, we recommend increasing the number of facilities where PrEP is available, including integrating PrEP into routine SRH services, and ensuring a consistent and reliable supply of PrEP by engaging with the Department of Health [33].

Limitations to this study include low sample realisation, due to some programme beneficiaries being uncontactable via phone; as a consequence, results of the study may not be representative of all programme beneficiaries. Nevertheless, sample realisation was similar to other telephonic surveys among AGYW in sub-Saharan Africa [47]. Given that participants had to have access to a phone to participate, a bias may have been introduced into results, as poorer and more vulnerable participants could have been excluded. However, recent data from an in-person baseline survey of 2,377 AGYW in school in 2 provinces in South Africa, where a large-scale combination HIV prevention programme is going to be implemented this year, found that only 10.7% of AGYW did not have their own phone or access to someone else’s phone [48]. In terms of our analyses, we included all AGYW who had sex in the past 6 months in our population in need despite the potential variability in HIV risk among this group because evidence from the baseline survey suggested that AGYW who had ever had sex were more likely to be living with HIV and any further specification of this group would have limited our sample size. It is possible that some AGYW living with HIV were included in our analyses given that HIV status was self-reported, but we do not think that this is very likely as all questions had a “prefer not to answer” option. In addition, the small effect size of certain factors included in our analyses may have caused some potential barriers to condom and PrEP use to be missed, but this would not affect the relationships that were reported as statistically significant. Finally, our dataset did not include certain potential barriers to condom and PrEP use including social norms, peer and parental influence, intimate partner violence, and access to youth friendly SRH information and services; these should be explored in future studies [40, 43, 46].

## Conclusion

Lack of effective use of condoms and lack of access to PrEP were major obstacles to preventing HIV infection among a random sample of AGYW enrolled in the My Journey programme in South Africa. The My Journey programme and similar programmes should focus on risk-reduction counselling for AGYW and their male partners to improve effective use of condoms, ensure condoms and PrEP are available at a wide-range of facilities which are easily accessible to young people and limit PrEP stock-outs by negotiating a consistent supply of PrEP from government. These findings demonstrate the use of HIV prevention cascades to measure the steps and barriers to HIV prevention in the context of prevention programmes in low-resourced countries and highlight important indicators that should be included in routine programme data. Comparing HIV prevention cascades like those described in this study over time could be a useful way to monitor and evaluate whether programmes are effectively increasing motivation, access and effective use of HIV prevention methods. Researchers and programme managers should consider creating a combined cascade of condom and PrEP use to see if participants are effectively using either prevention method in future studies with higher PrEP uptake.

### Supplementary Information

Below is the link to the electronic supplementary material.

Supplementary file1 (DOCX 90 KB)
