# Supplementary material for: Applying the HIV Prevention Cascade to an Evaluation of a Large-Scale Combination HIV Prevention Programme for Adolescent Girls and Young Women in South Africa
Source: AIDS Behav. 2023 Jul 18;28(4):1137–51. doi: 10.1007/s10461-023-04130-z (PMC10940416; doi:10.1007/s10461-023-04130-z)
Supplement: Supplementary file 1 — Supplementary file1 (DOCX 90 KB) [file 10461_2023_4130_MOESM1_ESM.docx]

# **Supplementary information**

**Box S1. Items included in the relative socio-economic status (SES) measure**

| 1. AGYW was away from home for more than one month in past 12 months (internal migration has been shown to cause and be caused by poverty (1))  2. Has piped water in household  3. Has flushing toilet in household  4. Household has working electricity  5. Household has a car  6. Household has a computer  7. Household has the internet  8. Household has a refrigerator  9. Household has a stove  10. AGYW or member of her household went a day/night without eating in the past month  11. AGYW has own money  12. AGYW saves money  13. AGYW owes money |
| --- |

**Box S2. Items included in the measure for not in education, employment or training (NEET)**

| 1. Are you still in school? (By school, we mean primary school or high school) 2. In 2020, before COVID-19 and the lockdown, were you enrolled in school, or college or university full-time? 3. In October 2020, were you enrolled in school, or college or university full-time? 4. Before COVID-19 and the lockdown, During January, February and March 2020, how often did you work to earn money? 5. In the past week, did you work to earn money? 6. In the past four weeks, how many days did you work to earn money? |
| --- |

**Table S1. Survey questions used for descriptive statistics and statistical analyses**

| **Question** | **Response** |
| --- | --- |
| When were you born? If you are not sure, please estimate your date of birth. | text |
| In the past 12 months have you been away from your usual home for more than one month? | Yes |
|  | No |
|  | Prefer not to answer |
| At home, do you have any of these things in working condition? Choose all the items that you have. | Electricity |
|  | Car |
|  | Computer/laptop |
|  | Internet |
|  | Fridge |
|  | Stove |
|  | TV |
|  | Radio |
| In the past four weeks, how often did you or any other member of your household go a whole day and night without eating because of lack of food? | Once |
|  | Twice |
|  | Three times |
|  | More than three times |
|  | Never |
|  | Prefer not to answer |
| Do you have your own money? | Yes |
|  | No |
|  | Prefer not to answer |
| Do you owe anyone money? | Yes |
|  | No |
|  | Prefer not to answer |
| Do you save money? (For example do you put money aside for another day or a special purpose?) | Yes |
|  | No |
|  | Prefer not to answer |
| During the lockdown I was not able to continue my schooling/studies. | Yes |
|  | No |
|  | Prefer not to answer |
| In 2020, before COVID-19 and the lockdown, were you enrolled in school, or college or university full-time? | I was enrolled in high school |
|  | I was enrolled in a TVET college |
|  | I was enrolled in another type of college |
|  | I was enrolled in university |
|  | I was enrolled in another training institution |
|  | I was not enrolled in any educational institution |
|  | Prefer not to answer |
| In October 2020, were you enrolled in school, or college or university full-time? | I was enrolled in high school |
|  | I was enrolled in a TVET college |
|  | I was enrolled in another type of college |
|  | I was enrolled in university |
|  | I was enrolled in another training institution |
|  | I was not enrolled in any educational institution |
|  | Prefer not to answer |
| Before COVID-19 and the lockdown, During January, February, and March 2020, how often did you work to earn money? | A few days |
|  | A few weeks |
|  | A few months |
|  | Every month |
|  | I did not work to earn money |
|  | Prefer not to answer |
| In the past week, did you work to earn money? | No I did not work to earn money |
|  | Yes, I worked a few hours during the week |
|  | Yes, I worked about 1 day of the week |
|  | Yes, I worked about 2 days of the week |
|  | Yes, I worked about 3 days of the week |
|  | Yes, I worked about 4 days of the week |
|  | Yes, I worked about 5 days or more of the week |
|  | No I lost my job or stopped wo COVID-19 and the lockdown |
|  | Prefer not to answer |
| In the past four weeks, how many days did you work to earn money? | I did not work |
|  | About 1 to 4 days |
|  | About 5 to 9 days |
|  | About 10 to 14 days |
|  | About 15 to 19 days |
|  | About 20 days or more |
|  | Prefer not to answer |
| Is your biological/birth mother alive? | Yes |
|  | No |
|  | I don’t know |
|  | Prefer not to answer |
| Is your biological/birth father alive? | Yes |
|  | No |
|  | I don’t know |
|  | Prefer not to answer |
| How old were you when you first had sex with a boy or man? Sex is when the penis enters the vagina or anus/bum. Sex can be something you agreed to, something you did not want to do, or were forced to do. | 11 years old or younger |
|  | 12 years old |
|  | 13 years old |
|  | 14 years old |
|  | 15 years old |
|  | 16 years old |
|  | 17 years old |
|  | 18 years old |
|  | 19 years old |
|  | 20 years old |
|  | 21 years old |
|  | 22 years old |
|  | 23 years old |
|  | 24 years old |
|  | I have not yet had sex |
|  | Prefer not to answer |
| Have you ever had an HIV test? | Yes |
|  | No |
|  | Prefer not to answer |
| Are you HIV positive / living with HIV? | Yes I am HIV positive |
|  | No I am HIV negative |
|  | I don’t know my status |
|  | Prefer not to answer |
| Have you ever been pregnant? | Yes |
|  | No |
|  | Unsure |
|  | Prefer not to answer |
| Have you ever given birth to a child? | Yes |
|  | No |
|  | Prefer not to answer |
| How many different boyfriends or male sexual partners have you had in the last 6 months? | text |
| In the past 6 months, have you had sex with a man who was older than you by 5 years or more? | Yes |
|  | No |
|  | Prefer not to answer |
| In the past six months, did you have sex (oral, vaginal or anal) with anyone to pay for the things you need? | Yes |
|  | No |
|  | Prefer not to answer |
| In the past 6 months, how often have you been afraid of boyfriend or partner or ex-boyfriend? | Never |
|  | Once |
|  | More than once |
|  | I have never had a boyfriend or partner |
|  | Prefer not to answer |
| How often do you drink 6 or more drinks on one occasion? | Never |
|  | Less than monthly |
|  | Weekly |
|  | Daily or almost daily |
|  | I never drink |
|  | Prefer not to answer |
| Did you have an HIV test in the past 6 months? | Yes |
|  | No |
|  | Prefer not to answer |
| If condoms were freely available, would you definitely want to use them? | Definitely |
|  | Probably |
|  | No opinion |
|  | Probably not |
|  | Definitely not |
|  | Prefer not to answer |
| If you want to get male condoms, how easy is it for you to get them? | Very easy |
|  | Easy |
|  | Unsure |
|  | Difficult |
|  | Very difficult |
|  | Prefer not to answer |
| We know that it is common for people to not use a condom every time. Think about the last boy or man you had sex with. What percent of the time did you use condoms with this person? (male or female condoms) If you said 0%, it means you did not use a condom with this partner. If you said 50% that means you used condoms about half the time and if you said 100% it means you used condoms every time you had sex with your partner. You can select any %. | Percentage (%) |
| If PrEP was freely available to you, would you definitely want to use it? | Definitely |
|  | Probably |
|  | No opinion |
|  | Probably not |
|  | Definitely not |
|  | Prefer not to answer |
| If you want to take PrEP, how easy would it be for you to get to a place where PrEP is provided? | Very easy |
|  | Easy |
|  | Neither easy nor difficult |
|  | Difficult |
|  | Very difficult |
|  | Prefer not to answer |
| If a person is HIV negative, by how much do you think male condoms reduce the person's risk of getting HIV when they have sex with someone who has HIV? You can answer anywhere from 0% to 100%. If you said 0% that would mean you do not think condoms reduce the risk of HIV. If you said 50% that would mean you think condoms are effective at reducing HIV risk half of the time. If you said 100% it means you think condoms are 100% effective at reducing HIV risk. | Percentage (%) |
| Why did you not use condoms 100% of the time when you had sex in the past three months? Choose as many answers as are relevant to you. | Forgot to use |
|  | I did not have condoms |
|  | The place I get my condoms is far away |
|  | The place I get my condoms was not open when I had free time |
|  | Because of the negative attitudes of health workers who give me condoms |
|  | I was worried about what my partner would think if I asked to use condoms |
|  | My sexual partner did not want me to use condoms |
|  | I have one faithful partner who I trust |
|  | I do not like using condoms |
|  | There was a stock-out and they did not have condoms for me |
|  | I do not think I am at risk of getting HIV |
|  | Other |
|  | Don't know the reason |
|  | I used condoms all of the time |
|  | I did not have sex in the past 3 months |
|  | Prefer not to answer |
| What makes it difficult for a person like you to get male condoms? Choose as many answers as apply. | Lack of privacy and confidentiality when getting them |
|  | Embarrassed to get them |
|  | It is expensive to get them |
|  | The place where I can get them is not open when I have time to go |
|  | It is far to travel for me to go get them |
|  | I am worried someone will see me getting them |
|  | Other |
|  | Nothing makes it difficult |
|  | Prefer not to answer |
| If I asked him to use a condom, he would get angry | Strongly disagree |
|  | Disagree |
|  | Agree |
|  | Strongly agree |
|  | I've never had a male boyfriend/partner |
|  | Refuse to answer |
| You were unable to get condoms because of COVID-19 or the lockdown | Never |
|  | Sometimes |
|  | Often |
|  | I did not need condoms |
|  | Prefer not to answer |
| A while back, someone from the organization (NGO) in your community contacted you to ask you if we could phone you and invite you to participate in this research. IN THE PAST MONTH, has anyone in that organization provided you with any of the following services, or linked you to people who provided you with these services? Choose as many as apply. | Condoms |
|  | HIV testing |
|  | Family planning |
|  | PrEP |
|  | HIV treatment |
|  | Help from a social worker |
|  | Help with another issue |
|  | They did not provide me with any services in the past month |
| Have you ever had instructions or counselling on how to use male condoms? | Yes |
|  | No |
|  | Prefer not to answer |
| The following statement is correct. Did you know this already?  "Pre-exposure prophylaxis (PrEP) is when someone who does not have HIV takes a pill on an ongoing basis to prevent them getting HIV. Most people who take PrEP take a pill everyday. PrEP needs to be taken for seven days before sex to be effective" | I know this already |
|  | I wasn't sure about this |
|  | I didn't know this |
|  | I do not understand this |
|  | Prefer not to answer |
| What makes it difficult or unsuitable for young woman like you to go to a place to get PrEP? Choose as many answers as are relevant. | It will cost too much to get to the clinic/service |
|  | I worry about lack of privacy or confidentiality |
|  | The opening hours of the clinic/service do not suit me |
|  | It is far to go to the clinic/service |
|  | I am not sexually active at the moment |
|  | I worry about people thinking I am HIV positive |
|  | The negative attitudes of the health workers |
|  | I am HIV positive |
|  | I don't need it |
|  | I don't know about it |
|  | Prefer not to answer |
| I am confident I can use PrEP if I wanted to | Strongly disagree |
|  | Disagree |
|  | Unsure |
|  | Agree |
|  | Strongly agree |
|  | Refuse to answer |
| I am confident I can use PrEP even if I have to take it every day | Strongly disagree |
|  | Disagree |
|  | Unsure |
|  | Agree |
|  | Strongly agree |
|  | Refuse to answer |
| I am confident I can use Prep even if I have to take it always after a meal | Strongly disagree |
|  | Disagree |
|  | Unsure |
|  | Agree |
|  | Strongly agree |
|  | Refuse to answer |
| I am confident I can use PrEP even if I have to hide it from my partner | Strongly disagree |
|  | Disagree |
|  | Unsure |
|  | Agree |
|  | Strongly agree |
|  | Refuse to answer |
| I am confident I can use PrEP even if my friends disapprove of this | Strongly disagree |
|  | Disagree |
|  | Unsure |
|  | Agree |
|  | Strongly agree |
|  | Refuse to answer |
| I am confident I can use PrEP even if my parents and family elders disapprove | Strongly disagree |
|  | Disagree |
|  | Unsure |
|  | Agree |
|  | Strongly agree |
|  | Refuse to answer |
| I am confident I can use PrEP even if people think I have HIV | Strongly disagree |
|  | Disagree |
|  | Unsure |
|  | Agree |
|  | Strongly agree |
|  | Refuse to answer |

**Table S2. Survey sample realisation and response rates by district, age and school status**

| **District** | **Bojanala** | **Klipfontein** | **King Cetshwayo** | **Ehlanzeni** | **Nelson Mandela Bay** | **Thabo Mofutsany ana** | **Total** |
| --- | --- | --- | --- | --- | --- | --- | --- |
| **N (%)** | **N (%)** | **N (%)** | **N (%)** | **N (%)** | **N (%)** | **N (%)** | **N (%)** |
| Principal Recipient | NACOSA | NACOSA | AFSA | AFSA | Beyond Zero | Beyond Zero |  |
| AGYW 15-19 years in school | 24/200 (12.0%) | 6/200 (3.0%) | 43/200 (21.5%) | 79/200 (39.5%) | 30/200 (15.0%) | 30/200 (15.0%) | **212/1200 (17.7%)** |
| AGYW 15-19 years out of school | 9/40 (22.5%) | 9/40 (22.5%) | 15/40 (37.5%) | 1/40 (2.5%) | 5/40 (12.5%) | 13/40 (32.5%) | **52/240 (21.7%)** |
| AGYW 20-24 years | 30/120 (25.0%) | 43/120 (35.8%) | 68/120 (56.7%) | 28/120 (23.3%) | 35/120 (29.2%) | 47/120 (39.2%) | **251/720 (34.9%)** |
| **Total AGYW** | **63/360 (17.5%)** | **58/360 (16.1%)** | **126/360 (35.0%)** | **108/360 (30.0%)** | **70/360 (19.4%)** | **90/360 (25.0%)** | **515/2160 (23.8%)** |

**Table S3: Weighted univariate and multivariable analysis of factors associated with motivation to use male condoms among AGYW who were HIV-negative and had sex in the past six months (n = 301)**

| **Barrier** | **Motivated to use male condoms N (%)** | **Motivated to use male condoms N(%) (weighted)** | | **Crude odds ratio (COR) (95% CI)** | | **Crude odds ratio (COR) (95% CI) (weighted)** | | **Adjusted odds ratio (aOR) (95% CI)** | | **Adjusted odds ratio (aOR) (95% CI) (weighted)** |
| --- | --- | --- | --- | --- | --- | --- | --- | --- | --- | --- |
| Age group (n = 301) | | | | | | | | | | |
| 15-19 | 92 (85.2) | 108.3 (86.7) | | - | | - | | - | | - |
| 20-24 | 169 (87.6) | 158.8 (90.2) | | 1.22 (0.62-2.42) | | 0.86 (0.64-3.16) | |  | |  |
| Relative SES group (out of four levels) (n = 300) | | | | | | | | | | |
|  | - | - | | 0.95 (0.69-1.30) | | 0.95 (0.69-1.32) | | - | | - |
| AGYW NEET in 2020 (n = 301) | | | | | | |  | |  | |
| No | 227 (87.3) | 230.9 (89.2) | | - | | - | | - | | - |
| Yes | 34 (82.9) | 36.2 (85.6) | | 0.71 (0.29-1.72) | | 0.72 (0.28-1.85) | |  | |  |
| **Knowledge of intervention** | | |  | |  | |  | |  | |
| AGYW did not think that male condoms reduce an HIV-negative person’s risk of getting HIV by 70% or more when they have sex with someone who has HIV (n = 298) | | | | | | | | | | |
| Believed | 176 (87.6) | 181.8 (88.9) | | - | | - | | - | | - |
| Did not believe | 82 (84.5) | 82.1 (87.7) | | 0.78 (0.39-1.55) | | 0.89 (0.39-2.04) | |  | |  |
| **HIV risk perception** | | | | | |  | |  | |  |
| AGYW did not think she was at risk of getting HIV (n = 301) | | | | | | | | | | |
| No | 255 (87) | 259.7 (88.8) | | - | | - | | - | | - |
| Yes | 6 (75) | 7.4 (87.4) | | 0.45 (0.09-2.30) | | 0.87 (0.13-5.97) | |  | |  |
| AGYW has one faithful partner who she trusts (n = 301) | | | | | | | | | | |
| No | 194 (90.2) | 196.6 (90.0) | | - | | - | | - | | - |
| Yes | 67 (77.9) | 70.5 (85.4) | | **0.38 (0.19**-**0.75)**** | | 0.65 (0.29-1.47) | | **0.44 (0.22**-**0.90)*** | | 0.81 (0.35-1.89) |
| **Consequences of use / attitudes** | | | | | | | | | | |
| AGYW does not like using condoms (n = 301) | | | | | | | | | | |
| No | 236 (89.7) | 238.5 (91) | | - | | - | | - | | - |
| Yes | 25 (65.8) | 28.6 (73.6) | | **0.22 (0.10**-**0.48)**** | | **0.28 (0.11**-**0.73)**** | | **0.26 (0.11**-**0.57)**** | | **0.29 (0.10**- **0.79)*** |
| AGYW agrees or strongly agrees that if she asked her current or most recent main partner/boyfriend to use a condom, he would get angry (n = 300) | | | | | | | | | | |
| Disagree | 224 (86.8) | 225.9 (88.5) | | - | | - | | - | | - |
| Agree | 36 (85.7) | 40.1 (89.8) | | 0.91 (0.36-2.32) | | 1.15 (0.34-3.94) | |  | |  |
| AGYW is embarrassed to get male condoms (n = 301) | | | | | | | | | | |
| No | 152 (88.9) | 176.2 (91.1) | | - | | - | | - | | - |
| Yes | 109 (83.8) | 81.5 (85.6) | | 0.65 (0.33-1.26) | | 0.53 (0.24-1.18) | |  | |  |
| AGYW is worried someone will see her getting male condoms (n = 301) | | | | | | | | | | |
| No | 163 (87.6) | 185.6 (91.1) | | - | | - | | - | | - |
| Yes | 98 (85.2) | 81.5 (84.5) | | 0.81 (0.41-1.60) | | 0.65 (0.29-1.46) | |  | |  |

Bold=p-value$\leq$0.05; *p-value < 0.05; **p-value < 0.01

**Table S4. Weighted univariate and multivariable analysis of factors associated with access to male condoms among AGYW who were motivated to use male condoms, HIV-negative and had sex in the past six months (n = 260)**

| **Barrier** | **Had access to male condoms N (%)** | **Had access to male condoms N (%) (weighted)** | **Crude odds ratio (OR) (95% CI)** | **Crude odds ratio (OR) (95% CI) (weighted)** | **Adjusted odds ratio (aOR) (95% CI)** | **Adjusted odds ratio (aOR) (95% CI) (weighted)** |
| --- | --- | --- | --- | --- | --- | --- |
| Age group (n = 260) | | | | | |  |
| 15-19 | 76 (82.6) | 88.9 (84.1) | - | - | - | - |
| 20-24 | 152 (90.5) | 140.5 (91.1) | 2 (0.95-4.22) | 1.93 (0.81-4.59) | 2.33 (0.95-5.70) | 1.93 (0.80-4.64) |
| Relative SES group (n = 260) | | | | | | |
|  | - | - | 1.14 (0.81-1.62) | 1.12 (0.75-1.68) | - | - |
| AGYW NEET in 2020 (n = 260) | | | | | | |
| No | 199 (88.1) | 199.7 (88.9) | - | - | - | - |
| Yes | 29 (85.3) | 29.7 (84.1) | 0.79 (0.28-2.21) | 0.66 (0.21-2.1) |  |  |
| **Availability** |  |  |  |  |  |  |
| AGYW did not have condoms (n = 260) | | | | | | |
| No | 175 (86.6) | 176.6 (87.1) | - | - | - | - |
| Yes | 53 (91.4) | 52.8 (92.3) | 1.64 (0.6-4.46) | 1.77 (0.61-5.13) |  |  |
| AGYW reported that there was a stock-out and they did not have condoms for her (n = 260) | | | | | | |
| No | 224 (88.2) | 224.2 (88.5) | - | - | - | - |
| Yes | 4 (66.7) | 5.2 (79.5) | 0.27 (0.05-1.53) | 0.51 (0.06-4.11) |  |  |
| **Accessibility** |  |  |  |  |  |  |
| AGYW was sometimes or often unable to get male condoms because of COVID-19 or the lockdown (n = 211) | | | | | | |
| No | 133 (89.9) | 121.7 (89.5) | - | - | - | - |
| Yes | 55 (87.3) | 67.3 (89.7) | 0.78 (0.31-1.93) | 1.02 (0.37-2.84) |  |  |
| In the past month, someone from an organisation involved in this research has provided the AGYW with condoms or linked her to people who could provide them (n = 260) | | | | | | |
| No | 188 (87.0) | 192.1 (88.1) | - | - | - | - |
| Yes | 40 (90.9) | 37.3 (89.1) | 1.49 (0.49-4.48) | 1.11 (0.32-3.85) |  |  |
| The place where AGYW gets her condoms was not open when she had free time (n = 260) | | | | | | |
| No | 217 (88.9) | 216.1 (88.9) | - | - | - | - |
| Yes | 11 (68.8) | 13.3 (79.0) | **0.27 (0.09-0.85)*** | 0.47 (0.14-1.61) |  |  |
| The place where AGYW gets her condoms is far away (n = 260) | | | | | | |
| No | 194 (90.2) | 182.4 (90.3) | - | - | - | - |
| Yes | 34 (75.6) | 47.0 (81.0) | **0.33 (0.15-0.76)**** | 0.46 (0.18-1.17) | **0.25 (0.10**-**0.64)**** | 0.46 (0.18-1.19) |
| **Acceptability** |  |  |  |  |  |  |
| AGYW finds it difficult to get male condoms because of the lack of privacy and confidentiality when getting them (n = 260) | | | | | | |
| No | 181 (89.6) | 183.2 (89.6) | - | - | - | - |
| Yes | 47 (81.0) | 46.2 (83.3) | 0.5 (0.22-1.10) | 0.58 (0.22-1.54) |  |  |
| AGYW reported that the negative attitudes of health workers who give her condoms is a barrier (n = 260) | | | | | | |
| No | 219 (88.3) | 219.3 (88.9) | - | - | - | - |
| Yes | 9 (75.0) | 10.1 (76.4) | 0.4 (0.10-1.55) | 0.4 (0.07-2.21) |  |  |
| **Affordability** |  |  |  |  |  |  |
| AGYW finds it difficult to get male condoms because it is expensive to get them (n = 260) | | | | | | |
| No | 217 (88.6) | 217.2 (88.8) | - | - | - | - |
| Yes | 11 (73.3) | 12.2 (78.6) | 0.35 (0.11-1.19) | 0.46 (0.13-1.67) |  |  |

Bold=p-value$\leq$0.05; *p-value < 0.05; **p-value < 0.01

**Table S5: Weighted univariate and multivariable analysis of factors associated with effective use of condoms among AGYW who had access to male condoms, were motivated to use male condoms, were HIV-negative and had sex in the past six months (N = 223)**

| **Barrier** | **Effectively used condoms N (%)** | **Effectively used condoms N (%) (weighted)** | **Crude odds ratio (OR) (95% CI)** | | **Crude odds ratio (OR) (95% CI) (weighted)** | | **Adjusted odds ratio (aOR) (95% CI)** | | **Adjusted odds ratio (aOR) (95% CI) (weighted)** |
| --- | --- | --- | --- | --- | --- | --- | --- | --- | --- |
| Age group (n = 223) | | | | | | |  | |  |
| 15-19 | 15 (20.0) | 18.6 (21.1) | - | | - | | - | | - |
| 20-24 | 27 (18.2) | 26.2 (19.4) | 0.89 (0.44-1.8) | | 0.90 (0.40-2.04) | |  | |  |
| Relative SES group (n = 223) | | | | | | | |  | |
|  |  |  | 1.1 (0.81-1.51) | | 1.12 (0.75-1.68) | | - | | - |
| AGYW NEET in 2020 (n = 223) | | | | | | | |  | |
| No | 36 (18.5) | 40.1 (20.7) | - | | - | | - | | - |
| Yes | 6 (21.4) | 4.7 (16.2) | 1.2 (0.46-3.19) | | 0.74 (0.26-2.14) | |  | |  |
| AGYW has six or more drinks on one occasion every month or more (n = 223) | | | | | | | | | |
| Never | 24 (17.6) | 29.0 (20.9) | - | | - | | - | | - |
| Every month or more frequently | 17 (19.8) | 15.4 (18.5) | 1.15 (0.58-2.29) | | 0.86 (0.39-1.86) | |  | |  |
| **Skills** |  |  |  | |  | |  | |  |
| AGYW has had instructions or counselling on how to use male condoms (n = 223) | | | | | | | | | |
| No | 13 (14.1) | 11.4 (12.5) | - | | - | | - | | - |
| Yes | 29 (22.1) | 33.4 (25.3) | 1.73 (0.84-3.54) | | 2.37 (0.97-5.77) | | **2.24 (1.05**-**4.76)*** | | **2.75 (1.12**-**6.76)*** |
| **Partner influence / refusals** | | | |  | |  | |  | |
| AGYW’s sexual partner does not want her to use condoms (n = 223) | | | | | | | | |  |
| No | 41 (20.7) | 43.6 (22.3) | - | | - | | - | | - |
| Yes | 1 (4.0) | 1.2 (4.2) | 0.16 (0.02-1.21) | | 0.15 (0.02-1.2) | | 0.18 (0.02-1.38) | | 0.15 (0.02-1.22) |
| In the past six months, AGYW had sex (oral, vaginal or anal) with someone to pay for the things she needs (n = 223) | | | | | | | | | |
| No | 42 (19.7) | 44.8 (21.4) | Omitted | | Omitted | | Omitted | | Omitted |
| Yes | 0 (0.0) | 0.0 (0.0) |  | |  | |  | |  |
| In the past 6 months, AGYW had sex with a man who was older than her by 5 years or more (n = 223) | | | | | | | | |  |
| No | 36 (22) | 39.5 (23.3) | - | | - | | - | | - |
| Yes | 6 (10.2) | 5.3 (9.9) | **0.4 (0.16-1.01)** | | **0.36 (0.14-0.96)*** | | 0.41 (0.16-1.06) | | **0.34 (0.12**-**0.92)*** |
| In the past 6 months, AGYW was afraid of her partner (n = 223) | | | | | | | | | |
| Less often or never | 40 (19.3) | 40.6 (19.6) | - | | - | | - | | - |
| More than once | 2 (12.5) | 4.2 (26.9) | 0.6 (0.13-2.73) | | 1.52 (0.32-7.22) | |  | |  |

Bold=p-value$\leq$0.05; *p-value < 0.05; **p-value < 0.01

**Table S6: Weighted univariate and multivariable analysis of factors associated with motivation to use PrEP among AGYW who were HIV-negative, had sex in the past six months and had never taken PrEP (n = 270)**

| **Barrier** | **Motivated to use PrEP N (%)** | **Motivated to use PrEP N (%) (weighted)** | **Crude odds ratio (OR) (95% CI)** | **Crude odds ratio (OR) (95% CI) (weighted)** | **Adjusted odds ratio (aOR) (95% CI)** | | | **Adjusted odds ratio (aOR) (95% CI) (weighted)** |
| --- | --- | --- | --- | --- | --- | --- | --- | --- |
| Age group (n = 270) | | | |  |  | | |  |
| 15-19 | 74 (74.7) | 80.5 (70.3) | - | - | - | | | - |
| 20-24 | 131 (76.6) | 119.4 (76.8) | 1.11 (0.62-1.97) | 1.40 (0.73-2.68) |  | | |  |
| Relative SES group (n = 270) | | | | | | |  | |
|  |  |  | 0.95 (0.69-1.3) | 1.02 (0.74-1.41) | - | | | - |
| AGYW NEET in 2020 (n = 270) | | | | | | | | |
| No | 176 (75.2) | 170.2 (73.6) | - | - | - | | | - |
| Yes | 29 (80.6) | 29.7 (76.6) | 1.37 (0.57-3.28) | 1.17 (0.44-3.12) | 4.6 (1.15-18.35)* | | | 2.58 (0.89-7.52) |
| **Knowledge of intervention** | | | | | | | | |
| AGYW knew about PrEP and was sure about what it was (n = 270) | | | | | | | | |
| No | 131 (77.1) | 126.6 (74.6) | - | - | - | | | - |
| Yes | 74 (74.0) | 73.2 (73.1) | 0.85 (0.48-1.50) | 0.93 (0.49-1.77) | **0.51 (0.24-1.06)** | | | 0.50 (0.23-1.09) |
| AGYW believed that PrEP could reduce a person’s risk of getting HIV by 70% or more (n = 260) | | | | | | | | |
| Believed | 139 (84.8) | 127.4 (82.9) | - | - | - | | | - |
| Did not believe | 60 (62.5) | 67.2 (63.1) | **0.30 (0.17-0.54)**** | **0.35 (0.18-0.70)**** | **0.35 (0.17-0.72)**** | | | **0.37 (0.18-0.79)*** |
| **HIV risk perception** |  |  |  |  |  | | |  |
| AGYW did not think she was at risk of getting HIV (n = 270) | | | | | | | | |
| No | 201 (76.1) | 197.7 (74.7) | - | - | - | | | - |
| Yes | 4 (66.7) | 2.1 (39.4) | 0.63 (0.11-3.5) | 0.22 (0.03-1.44) |  | | |  |
| AGYW has one faithful partner who she trusts (n = 270) | | | | | | | | |
| No | 141 (73.8) | 140.9 (72.1) | - | - | - | | | - |
| Yes | 64 (81.0) | 59.0 (79.1) | 1.51 (0.79-2.89) | 1.47 (0.67-3.21) |  | | |  |
| In the past six months, AGYW had sex (oral, vaginal or anal) with someone to pay for the things she needs (N = 270) | | | | | | | | |
| No | 195 (75.3) | 187.5 (73.4) | - | - | - | | | - |
| Yes | 10 (90.9) | 12.4 (85.4) | 3.28 (0.41-26.14) | 2.13 (0.25-17.86) |  | | |  |
| In the past 6 months, AGYW had sex with a man who was older than her by 5 years or more (n = 270) | | | | | | | |  |
| No | 151 (75.9) | 152.3 (74.2) | - | - | - | | | - |
| Yes | 54 (76.1) | 47.6 (73.5) | 1.01 (0.54-1.9) | 0.96 (0.47-1.98) |  | | |  |
| In the past 6 months, AGYW was afraid of her partner (n = 270) | | | | | |  | | |
| Less often or never | 189 (75.9) | 184.7 (73.5) | - | - | - | | | - |
| More than once | 16 (76.2) | 15.2 (80.4) | 1.02 (0.36-2.89) | 1.47 (0.46-4.71) |  | | |  |
| AGYW had an HIV test in the past six months (n = 257) | | | | | | | | |
| No | 40 (76.9) | 33.3 (70.9) | - | - | - | | | - |
| Yes | 157 (76.6) | 159.2 (75.8) | 0.98 (0.48-2.02) | 1.28 (0.55-3.00) |  | | |  |
| **Consequences of use / attitudes** | | | | | | |  | |
| AGYW reported that it is difficult to get to a place to get PrEP because she worries about people thinking she is HIV-positive (n = 270) | | | | | | | | |
| No | 130 (72.6) | 130.3 (71.4) | - | - | - | | | - |
| Yes | 75 (82.4) | 69.6 (79.5) | 1.77 (0.94-3.32) | 1.55 (0.76-3.17) | **2.28 (1.03-5.05)*** | | | 2.10 (0.84-5.26) |
| AGYW was confident she would be able to use PrEP if she wanted to (n = 270) | | | | | | | | |
| No | 37 (53.6) | 41.2 (53.6) | - | - | - | | | - |
| Yes | 168 (83.6) | 158.7 (82.1) | **4.40 (2.41-8.04)**** | **3.97 (1.99-7.93)**** | **2.99 (1.41-6.35)**** | | | **3.27 (1.53-6.98)**** |
| AGYW was confident she would be able to take PrEP every day (n = 270) | | | | | | | | |
| No | 48 (58.5) | 58.1 (60.9) | - | - | - | | | - |
| Yes | 157 (83.5) | 141.8 (81.2) | **3.59 (2.00-6.43)**** | **2.78 (1.43-5.43)**** |  | | |  |
| AGYW was confident she would always be able to take PrEP after a meal (n = 270) | | | | | | | | |
| No | 43 (56.6) | 45.0 (57.4) | - | - | - | | | - |
| Yes | 162 (83.5) | 154.8 (80.8) | **3.89 (2.15-7.02)**** | **3.12 (1.59-6.14)**** | **3.04 (1.37-6.75)**** | | | 1.97 (0.92-4.24) |
| AGYW was confident she would be able to use PrEP if she had to hide it from her partner (n = 270) | | | | | | | |  |
| No | 104 (72.2) | 101.3 (71.6) | - | - | - | | | - |
| Yes | 101 (80.2) | 98.5 (76.8) | 1.55 (0.88-2.75) | 1.31 (0.68-2.52) |  | | |  |
| GYW was confident she would be able to use PrEP if her friends disapproved of it (n = 270) | | | | | | | | |
| No | 34 (59.6) | 33.8 (59.5) | - | - | - | | | - |
| Yes | 171 (80.3) | 166.1 (77.9) | **2.75 (1.47-5.16)**** | **2.40 (1.17-4.92)*** |  | | |  |
| AGYW was confident she would be able to use PrEP if her parents and family elders disapproved (n = 270) | | | | | | | | |
| No | 52 (61.2) | 49.2 (58.7) | - | - | - | | | - |
| Yes | 153 (82.7) | 150.7 (80.9) | **3.03 (1.70-5.41)**** | **2.98 (1.53-5.81)**** | **1.93 (0.92-4.02)** | | | **2.27 (1.**11-4**.63)*** |
| GYW was confident she would be able to use PrEP if people thought she had HIV (n = 270) | | | | | | | | |
| No | 26 (54.2) | 27.3 (53.8) | - | - | - | | | - |
| Yes | 179 (80.6) | 172.5 (78.7) | **3.52 (1.82-6.80)**** | **3.18 (1.5-6.78)**** |  | | |  |

Bold=p-value$\leq$0.05; *p-value < 0.05; **p-value < 0.01

**Table S7: Weighted univariate and multivariable analysis of factors associated with access to PrEP among AGYW who were motivated to use PrEP, were HIV-negative, had sex in the past six months and had never taken PrEP (n = 205)**

|  | **Had access to PrEP N (%)** | **Had access to PrEP N (%) (weighted)** | **Crude odds ratio (OR) (95% CI)** | **Crude odds ratio (OR) (95% CI) (weighted)** | **Adjusted odds ratio (aOR) (95% CI)** | **Adjusted odds ratio (aOR) (95% CI) (weighted)** |
| --- | --- | --- | --- | --- | --- | --- |
| Age group (n = 205) | | | | | | |
| 15-19 | 35 (47.3) | 37.8 (45.8) | - | - | - | - |
| 20-24 | 78 (59.5) | 70.6 (57.7) | 1.64 (0.92-2.91) | 1.61 (0.81-3.21) |  |  |
| Relative SES group (n = 205) | | | | | | |
|  |  |  | 0.91 (0.70-1.18) | 0.91 (0.67-1.23) | - | - |
| AGYW NEET in 2020 (n = 205) | | | | | | |
| No | 93 (52.8) | 87.1 (49.9) | - | - | - | - |
| Yes | 20 (69.0) | 21.3 (70.0) | 1.98 (0.86-4.6) | 2.35 (0.92-6.01) |  |  |
| **Availability** |  |  |  |  |  |  |
| AGYW has ever been offered PrEP (n = 205) | | | | | | |
| No | 91 (51.7) | 85.6 (48.7) | - | - | - | - |
| Yes | 22 (75.9) | 22.8 (77.9) | **2.94 (1.19-7.22)*** | **3.72 (1.42-9.74)**** | **2.94 (1.19-7.22)*** | **3.72 (1.42-9.74)**** |
| **Accessibility** |  |  |  |  |  |  |
| AGYW believes the opening hours of the PrEP clinic/service would not suit her (n = 205) | | | | | | |
| No | 105 (55.6) | 97.8 (52.0) | - | - | - | - |
| Yes | 8 (50.0) | 10.6 (62.8) | 0.8 (0.29-2.22) | 1.56 (0.45-5.37) |  |  |
| AGYW believes it is far to go to the PrEP clinic/service (n = 205) | | | | | | |
| No | 102 (55.1) | 91.6 (51.9) | - | - | - | - |
| Yes | 11 (55.0) | 16.8 (58.9) | 0.99 (0.39-2.51) | 1.33 (0.47-3.77) |  |  |
| **Acceptability** |  |  |  |  |  |  |
| AGYW would worry about lack of privacy or confidentiality at a PrEP service (n = 205) | | | | | | |
| No | 84 (55.6) | 80.6 (55.3) | - | - | - | - |
| Yes | 29 (53.7) | 27.9 (47.0) | 0.93 (0.5-1.73) | 0.72 (0.34-1.50) |  |  |
| AGYW believes that the negative attitudes of the health workers at a PrEP clinic/service would make it difficult for her to get PrEP (n = 205) | | | | | | |
| No | 86 (52.8) | 83.7 (51.2) | - | - | - | - |
| Yes | 27 (64.3) | 24.7 (59.4) | 1.61 (0.80-3.25) | 1.39 (0.59-3.28) |  |  |
| **Affordability** |  |  |  |  |  |  |
| AGYW believes it would cost too much to get to the clinic/service to get PrEP (n = 205) | | | | | | |
| No | 107 (54.9) | 98.7 (51.6) | - | - | - | - |
| Yes | 6 (60.0) | 9.8 (70.2) | 1.23 (0.34-4.51) | 2.21 (0.47-10.3) |  |  |

Bold=p-value$\leq$0.05; *p-value < 0.05; **p-value < 0.01

**References**

1. Crush J, Frayne B. Surviving on the move: migration, poverty and development in southern Africa. In 2010.
